# Nano-porous Al/Au skeleton to support MnO_2_ with enhanced performance and electrodeposition adhesion for flexible supercapacitors†

**DOI:** 10.1039/d1ra01923f

**Published:** 2021-06-24

**Authors:** Du Huang, Zhenya Lu, Qian Xu, Xingyue Liu, Wenbin Yi, Junning Gao, Zhiwu Chen, Xin Wang, Xiaoyi Fu

**Affiliations:** School of Materials Science and Engineering, South China University of Technology Guangzhou 510641 China zhylu@scut.edu.cn

## Abstract

A nano-porous Al/Au skeleton is constructed to effectively improve the utilization rate of the active MnO_2_ and the overall adhesion between the current collector and MnO_2_ in an electrodeposition system. The Al/Au current collector is prepared by first forming a nano-porous structure on the surface of Al foil through etching modification, and subsequently coating an ultra-thin Au layer onto the Al foil. The active MnO_2_ is electrodeposited on the Al/Au current collector to fabricate a novel Al/Au/MnO_2_ electrode. The nano-porous skeleton supports MnO_2_ to grow autonomously inside-out. The ultra-thin Au layer acts as a transition layer to improve the overall conductivity of the current collector (0.35 Ω m^−1^) and to improve the adhesion with MnO_2_ as well. Owing to the highly porous structure, the electrochemical properties of the electrode are greatly improved, as evidenced by a remarkable specific capacitance of 222.13 mF cm^−2^ at 0.2 mA cm^−2^ and excellent rate capability of 63% capacitance retention at 6.0 mA cm^−2^. Furthermore, the assembled solid-state symmetric supercapacitor exhibits a high energy density of 0.68 mW h cm^−3^, excellent cyclic stability (86.3% capacitance retention after 2000 cycles), and prominent flexibility.

## Abstract 2

A nano-porous Al/Au skeleton is constructed to effectively improve the utilization rate of the active MnO_2_ and the overall adhesion between the current collector and MnO_2_ in an electrodeposition system.

## Graphical abstract

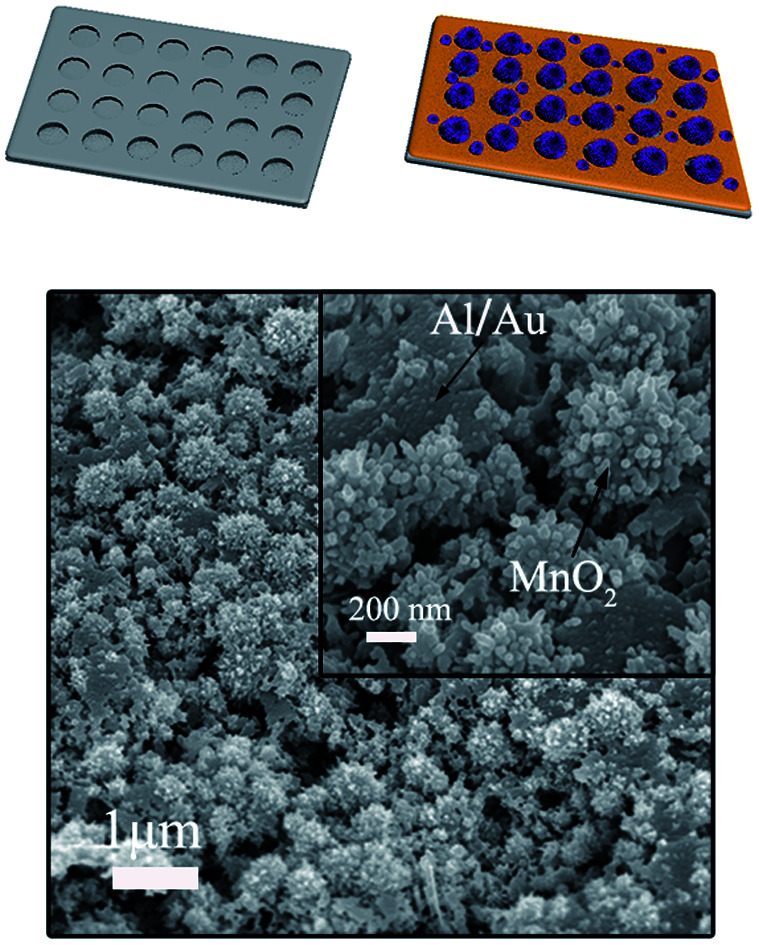

## 1. Introduction

As an energy supply element, supercapacitors have attracted widespread attention because of their unique energy storage mechanism and the characteristics of low equivalent series resistance, instantaneous high current, and long service life.^1–4^ Generally, there are two types of supercapacitors: electric double layer capacitors (EDLCs) formed by the reversible adsorption and desorption of ions and electrolytes, and pseudocapacitors formed by redox reaction and the Faraday charge–discharge process of active materials.^5^

A typical supercapacitor is generally composed of active material and a current collector, electrolyte, and separator. It is well acknowledged that the active materials provide energy storage capacity for supercapacitors. Therefore, in order to obtain electrodes with high specific capacitance, researchers put effort to grow various nanostructured active materials with high specific surface area (*S*_a_), such as nanotubes, nanospheres, and nanoflowers, *etc.*^6–11^ However, the synthesis methods for these structures are rather complicated, time-consuming. In fact, as the carrier for active materials, current collector is also a particularly crucial component, because the intrinsic characteristics of which determine the application scenarios of supercapacitors. In addition, some pseudocapacitive active materials with poor conductivity must be attached to the current collector to transmit electrons. Therefore, an ideal current collector should have high conductivity, strong adhesion with active materials, and good supporting stress. Common current collector materials include conductive metal (aluminum foil, copper foil, *etc.*), metal foam (foam nickel, foam aluminum, *etc.*), carbon substrate (carbon cloth, carbon paper, *etc.*) and some other conductive substrates. However, it is unfavorable to employ pristine metal substrate as current collector to support a mass of active materials, because the *S*_a_ of pristine metal is generally too low for insufficient contact area with the active material.^12^ Besides, the carbon cloth and the nickel foam are much more expensive than the metal foil. Thus, finding superior current collector materials remains challenging. Portet *et al.*^13^ reported a modification method of Al current collector that exhibited great surface contact and interface conductivity (0.4 Ω cm^−2^) between the carbon nanofibers. Huang *et al.*^14^ used picosecond laser scanning to construct hierarchical micro-nano patterned surface of Al current collector to increase surface area. Lang *et al.*^15^ developed a nano-porous gold current collector to support the growth of nanocrystalline MnO_2_, showing an extremely high specific capacitance. Meng *et al.*^16^ synthesized a polypyrrole (PPy)-decorated nano-porous gold (NPG) electrode with high volumetric capacitance. Kim *et al.*^17^ constructed a porous and oriented NiO–TiO_2_ nanotube arrays electrode by electrochemically anodizing Ni–Ti alloy foils. These methods were designed to prepare porous and high *S*_a_ current collectors for more active materials to grow, leading to improved specific capacitance. Another factor worth considering is the adhesion between current collector and active materials, for which affects the electrochemical performance and cyclic stability of supercapacitors. Kim *et al.*^18^ and Wang *et al.*^19^ discovered that coating graphene onto Cu and Al current collectors could better protect them from corrosion while improving the electrochemical properties, cyclic stability, and interlayer adhesion. It is therefore can be summarized that, from the perspective of perfecting the current collector, the performance of supercapacitor can be improved *via* (1) increasing its *S*_a_; (2) enhancing its conductivity and adhesion with active materials.

On the other hand, electronic devices are developing into the direction of flexibility and portability, so the research on flexible supercapacitors has also attracted much attention.^20^ The key to flexible supercapacitors is the flexible current collector and solid electrolyte.^21^ Aluminum (Al) foil is an ideal current collector material due to its high conductivity, good flexibility, stable mechanics and more importantly, low cost. Generally, there are two typical etching processes to increase the *S*_a_ of Al foil. The first option submerges the Al foil into the acid solution (chemical etching).^13^ The secondary option is electrolysis where the Al as the anode is placed in acid solution to form porous Al_2_O_3_ (AAO) layer (electrochemical etching).^22^ However, the preparation process for AAO is usually time and energy consuming. In overall consideration on the preparation process complexity, acid etching is chosen in this investigation. In previous works, researchers usually grew carbon-based materials on Al current collector to form double layer supercapacitor, but the specific capacitance of such electrodes only realized to ∼100 F g^−1^.^13,14^ In order to achieve a higher specific capacitance, transition metal oxide MnO_2_ was chosen to grow onto the Al current collector to prepare pseudocapacitive capacitors in this work. Electrodeposition is a common method of growing thin films, which has the advantages of simple, convenient and one-step operation. However, so far, no reports have been found about directly depositing MnO_2_ onto Al current collector by electrodeposition method. This may be due to the poor adhesion of Al itself or the aluminum oxide thin surface layer formed by oxidation of Al foil with MnO_2_ in the electrolyte of electrodeposition system. The specific mechanism is still unknown. Kavian *et al.*^23^ grew MWCNT felt onto Al foil first and then electrodeposited MnO_2_ to form composite electrode. However, the Al foil in the report was flat type which only acted as a role in transporting electrons. The MnO_2_ actually grown upon the CNT framework. It was also strange that in the control experiment, nickel sheets were selected to be current collector for electrodepositing MnO_2_ but not the original Al foil. So, maybe a transition layer was needed to grow onto the Al foil to enhance the overall adhesion between current collector and MnO_2_ if the electrodeposition was conducted.

In this work, the optimal conditions to form nano-porous structure of Al foil by etching modification were explored. In order to both utilize the nano-porous structure and the conductive property of etched Al foil, a large amount of coating and electrodeposition experiments had been explored, and we finally found that the adhesion between gold (Au) and Al or MnO_2_ was both good. Thereby, an ultra-thin Au layer was coated onto the Al foil to obtain Al/Au current collector. Then, the MnO_2_ could be successfully electrodeposited onto the Al/Au skeleton to form an Al/Au/MnO_2_ electrode. The nano-porous skeleton could support massive MnO_2_ to grow inside-out autonomously. Compared with the electrode prepared by pristine Al foil, the modified electrode exhibited superior electrochemical properties with a much higher specific capacitance, enhanced rate capability and better cyclic stability. Moreover, based on the Al/Au/MnO_2_ electrodes, the assembled symmetric solid-state supercapacitor presented excellent flexibility, high energy and power density.

## 2. Experiment

### 2.1 Surface modification of the Al foil

The pristine Al foil was cut into pieces (2.0 × 1.5 cm^2^) and then soaked in acetone and ethyl alcohol firstly to remove the surface grease. After that, the Al foils were immersed in a mixture acid of HCl and H_2_SO_4_ (HCl : H_2_SO_4_ = 1 : 1 M) at 80 °C for etching with different durations of 10, 20, 30 and 40 s with the products correspondingly named as A1, A2, A3 and A4, respectively. To investigate the effect of acid concentration, pristine Al foils were immersed also 30 s by changing the concentration of H_2_SO_4_ (HCl : H_2_SO_4_ = 1 : 2 M, HCl : H_2_SO_4_ = 1 : 3 M) at 80 °C, and named as A5, A6, respectively. After etching, the Al foils were thoroughly washed with ethyl alcohol and deionized water to remove any residual acid.

### 2.2 Preparation of the Al/Au/MnO_2_ electrode

An ultra-thin Au layer was coated onto the etched Al foil for about 5 min to form Al/Au current collector through an ion-coating machine (Eiko IB-3 ion coater). After that, MnO_2_ was electrodeposited directly onto the Al/Au current collector in a electrolyte including 0.1 M Mn(CH_3_COO)_2_·4H_2_O and 0.1 M Na_2_SO_4_ under a voltage of +0.8 V for 400 s. All depositions were proceeded with a three-electrode system at room temperature. The working, counter and reference electrode are respectively undertaken by Al/Au foil, Pt sheet and saturated calomel electrode (SCE). After deposition, the obtained Al/Au/MnO_2_ electrodes were washed with deionized water to remove the residual electrolyte and subsequently annealed at 80 °C for 30 min. The valid area of active MnO_2_ was 1.5 × 1 cm^2^. The thickness of the electrode is 0.03 mm. The mass of the samples before and after deposition were measured by a precise electronic balance. The mass loading of MnO_2_ was about 0.73 mg cm^−2^ (1.1 mg in 1.5 cm^2^). Electrodes prepared by pristine Al foils and A1 to A6 etched Al foils were denoted as AAM, AAM1 to AAM6, respectively.

### 2.3 Construction of all solid-state symmetric supercapacitors

The solid electrolyte was a mixture of 1.44 g Na_2_SO_4_ and 4 g PVA in 40 mL deionized water, which subjected to continuous stirring at 90 °C until becoming pellucid. An NKK (Nippon Kodoshi Corporation) separator and two electrodes were firstly immersed in the gel electrolyte for about 30 min, then the two electrodes were assembled face to face separating with Na_2_SO_4_/PVA gel and a separator to gain a solid-state symmetric supercapacitor with effective thickness of about 0.015 cm. Finally, the supercapacitor was heated at 60 °C for 20 min to solidify.

### 2.4 Material characterization

The morphologies and crystallographic information of the samples were characterized by field emission scanning electron microscopy (SEM, Merlin, Zeiss) and the attached energy-dispersive X-ray spectroscopy (EDS), X-ray diffraction (XRD, Smartlab, Rigaku), and Raman spectrometer (HJY LabRAM Aramis, HORIBA Jobin Yvon), respectively. The elemental chemical state and chemical composition of the materials were determined by X-ray photoelectron spectroscopy (XPS, Axis Ultra DLD, KratOs).

### 2.5 Electrochemical measurement

All the electrodepositions and electrochemical properties measurements including cyclic voltammetry (CV), galvanostatic charge/discharge (GCD) and electrochemical impedance spectroscopy (EIS) were proceeded on an electrochemical workstation (CHI 660E).

The areal capacitance (*C*_A_, F cm^−2^) of the electrodes and supercapacitors derived from CV and GCD are evaluated by eqn (1) and (2), respectively,1
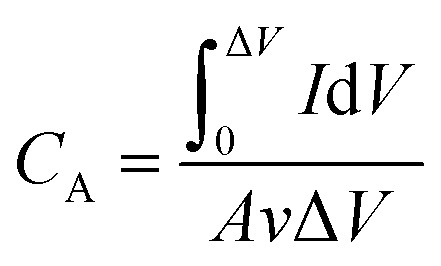
2
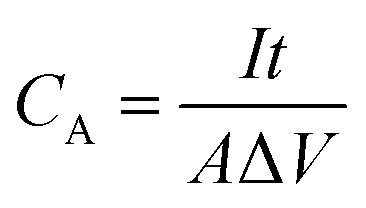
where 
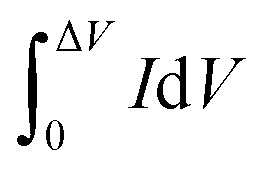
, *A* (cm^2^), *v* (V s^−1^), Δ*V* (V), *I* (A) and *t* (s) are the integral of the reduction area of the CV curve, valid area of active material, scan rate, potential range, current and discharging time, respectively.

The volumetric energy density (*E*, mW h cm^−3^) and power densities (*P*, mW cm^−3^) of the supercapacitors are evaluated by eqn (3) and (4), respectively,3
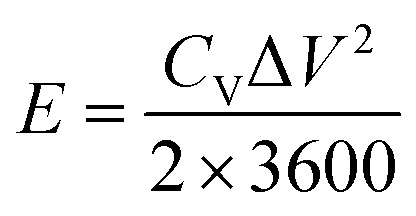
4
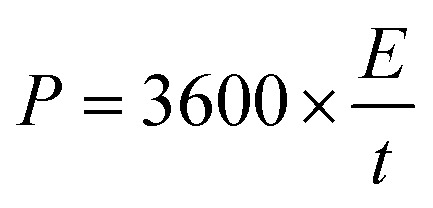
where Δ*V* (V), *C*_V_ (F cm^−3^) and *t* (s) represent cell voltage, the volumetric capacitance of supercapacitor and discharging time, respectively.

## 3. Results and discussion

### 3.1 Preparation and characterization of materials

**Fig. 1:**
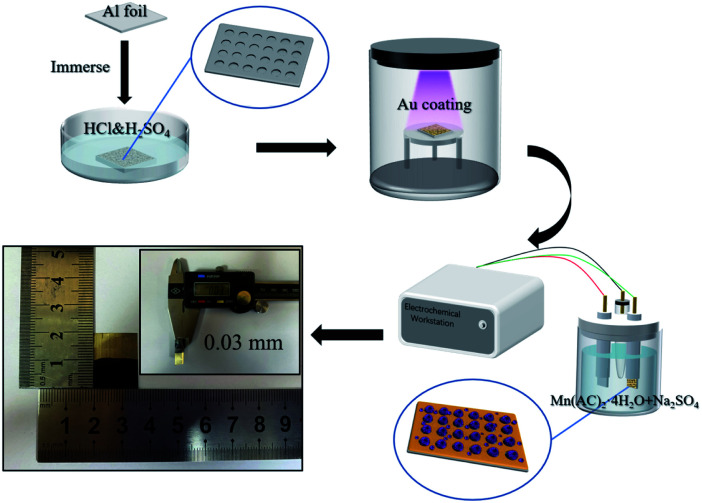
The fabrication procedure of Al/Au/MnO_2_ electrode.

**Fig. 2:**
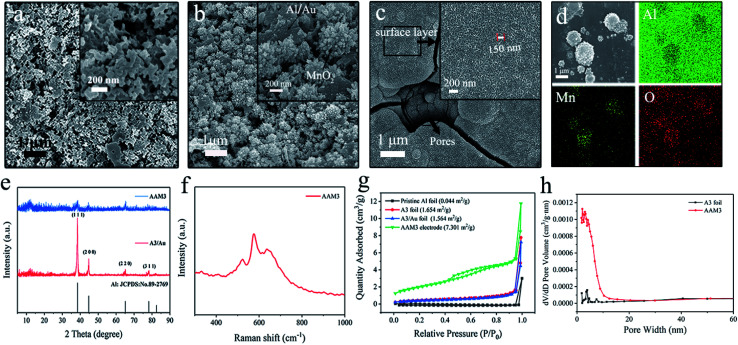
SEM images: (a) A3 foil; (b) internal and (c) surface of the as-prepared MnO_2_. (d) EDS mapping. (e) XRD patterns of the A3/Au current collector and the AAM3 electrode. (f) Raman spectra of AAM3 electrode. (g) N_2_ adsorption/desorption isotherms of the samples. (h) Pore size distribution of A3 foil and AAM3 electrode.

The fabrication procedure of Al/Au/MnO_2_ electrode is illustrated in Fig. 1. The SEM images of the surface morphologies for pristine Al foil and the etched Al foils (A1 to A6) under different corrosion conditions are presented in Fig. S1.† The surface of pristine Al foil is lackluster and smooth without pores, presenting a relatively small *S*_a_ (Fig. S1a†). After etching modification, the surface of A3 occurs abundant and uniform-distributed nano-pores and channels (Fig. 2a). The depth of these pores is about 200 nm. The SEM images of A1 and A2 foils with short etching durations (Fig. S1b and c†) show that the pores start to form at the beginning of the etching. However, these pores have not fully grown and distributed unevenly. With the prolonging etching time or the increasing of the concentration of H_2_SO_4_, pores grow rapidly and aggressively. The surface of A4, A5 and A6 foils is almost fully covered with bright stripes (Fig. S1d–f†). In the enlarged inset figures, the surface of Al foils is almost corroded into particles, and the emerged holes almost penetrate the Al foils (Fig. S1e and f†). These penetrating holes make the Al foil discontinuous and fragile, thus affecting its conductivity, and it is also difficult to support the growth of MnO_2_. Fig. S2† shows the uniform coverage of Au layer onto the surface of A3 foil to form the Al/Au current collector, which still maintains the nano-porous structure with high *S*_a_. Under electrodeposition, the MnO_2_ make full use of porous skeleton and grow from the pores and upon the surface simultaneously. These nano-structured MnO_2_ grows autonomously, presenting as nanoclusters or nanospheres in the pores (Fig. 2b) and as nanowires on the surface (Fig. 2c). The MnO_2_ nanowires with lengths about 150 nm are formed by the stacking of nanodots that are about 20–30 nm in diameter. The nano-porous current collector greatly increases the mass loading of MnO_2_, while the nanoscale MnO_2_ can fully contact with the electrolyte. These two combined effects make the AAM3 electrode gain a high *S*_a_ and improve the ions transmission rate in the electrolyte. The corresponding EDS mapping (Fig. 2d) and spectrum (Fig. S3†) reveal the distribution and composition of the electrode with Al, Mn and O elements, which demonstrates that the uniform distribution of MnO_2_ between nano-pores and the successful preparation of AAM3 electrode. To further study the growing process of MnO_2_, we also prepared electrodes base on A3/Au current collector under different deposition times (10 s, 20 s, 50 s, 100 s, 200 s, 300 s) with the same voltage of +0.8 V. The corresponding SEM images are shown in Fig. S4.† As the deposition time increases, the MnO_2_ grow from the pores inside-out and upon the surface simultaneously, then gradually fills the nano-pores and becomes a thicker surface MnO_2_ layer.

Fig. 2e displays the XRD patterns of A3/Au current collector and AAM3 electrode. It is obvious that all the diffraction peaks of A3/Au current collector can be indexed to Al (JCDPS 89-2769). Nevertheless, none of the peaks corresponds to manganese oxide, suggesting that the MnO_2_ prepared by electrodeposition is amorphous in nature which is consistent with previous report.^24^ Raman spectra of AAM3 electrode (Fig. 2f) exhibits peaks at 575 cm^−1^ accorded with Mn–O bond in the MnO_6_ octahedral and 638 cm^−1^ accorded with the Mn–O bond of the MnO_2_ framework,^25^ which also confirms the successful preparation of MnO_2_.

The specific surface areas of all the samples are measured by N_2_ adsorption and desorption test and shown in Fig. 2g. The curves correspond to a typical IV isotherm hysteresis loops.^26^ According to the BET analysis, the pristine Al foil exhibits a relatively small *S*_a_ of 0.044 m^2^ g^−1^. After etching modification, the *S*_a_ of A3 foil increase by nearly 40 times to 1.654 m^2^ g^−1^. When the ultra-thin Au layer is coated onto the A3 foil, the *S*_a_ can maintain 1.564 m^2^ g^−1^, which verify that the ultra-thin Au layer do not sacrifice the high *S*_a_ of nano-porous substrate. The AAM3 electrode presents a higher *S*_a_ of 7.301 m^2^ g^−1^. The pore size distribution (Fig. 2h) indicates the mesopores ranging from 2 to 8 nm of A3 foil and AAM3 electrode, which mainly attributes to the pores of current collector and the numerous gaps between MnO_2_ nanowires.

**Fig. 3:**
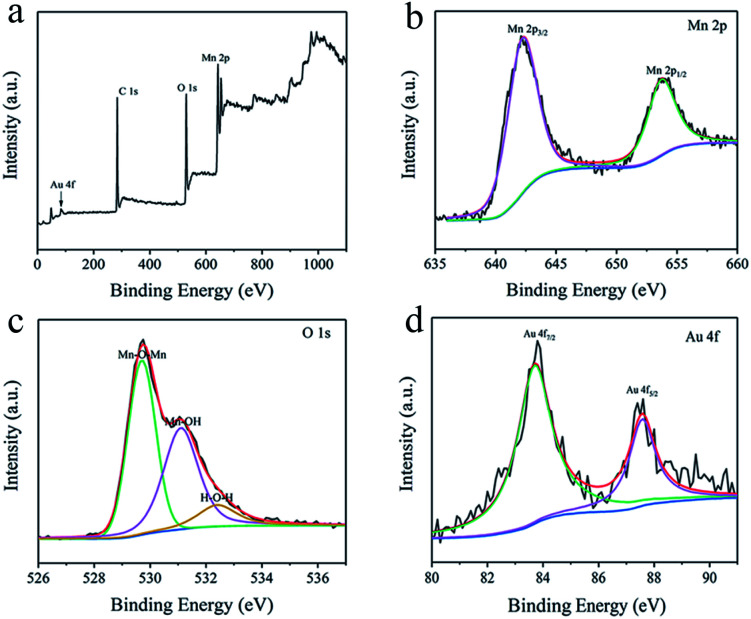
XPS spectra of the AAM3 electrode: (a) survey spectrum, (b) Mn 2p, (c) O 1s, and (d) Au 4f.

The XPS spectrums in Fig. 3 show the chemical states and composition of the AAM3 electrode. The XPS survey spectrum of the electrode indicates the existence elements of Au, Mn and O (Fig. 3a) which is consistent with the EDS results. The high-resolution Mn 2p spectrum (Fig. 3b) presents two obvious peaks at 642.2 and 653.8 eV, corresponding to Mn 2p_3/2_ and Mn 2p_1/2_ of Mn^4+^, agreeing with the previously reported MnO_2_ value.^27,28^ The high-resolution O 1s spectrum (Fig. 3c) presents three different peaks at 529.7, 531.1 and 532.4 eV, corresponding to Mn–O–Mn,^29^ Mn–O–H and water (H–O–H).^30^ Two peaks at 83.7 and 87.43 eV (Fig. 3d) corresponds to Au 4f_7/2_ and Au 4f_5/2_ core levels, respectively.^31^

The above analyses reveal the growing process and the uniform distribution of MnO_2_ from the nano-pores to the surface of the modified A3/Au current collector, also confirm the successful synthesis of nanoscale MnO_2_.

### 3.2 Electrochemical performances of the Al/Au/MnO_2_ electrode

**Fig. 4:**
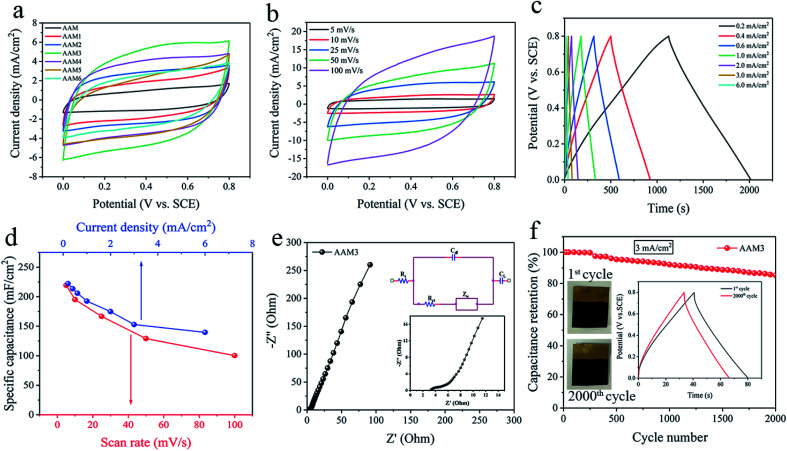
(a) The CV curves of AAM, AAM1 to AAM6 electrodes at the same scan rate of 25 mV s^−1^. Electrochemical behaviors of the AAM3 electrode: (b) CV curves from 5 to 100 mV s^−1^, (c) GCD curves from 0.2 to 6 mA cm^−2^, (d) the relationship between the specific capacitance and the scan rate or current density, (e) EIS Nyquist plots, with the insets showing the amplified Nyquist plots at high to medium frequencies and the equivalent circuit, (f) the GCD cyclic stability of 2000 cycles at 3 mA cm^−2^, with the inset showing the comparison of the first and the 2000^th^ cycle GCD.

The capacitance, charge–discharge and impedance characteristics of the prepared electrodes are evaluated by CV, GCD and EIS projects in 1 M Na_2_SO_4_ electrolyte. According to the guidance of electrochemical capacitors in previous reports, the electrochemical performances of electrode are evaluated in areal values.^32,33^ The CV curves of AAM, AAM1 to AAM6 electrodes with the same scan rate of 25 mV s^−1^ are shown in Fig. 4a. Obviously, AAM3 electrode presents the largest CV loop among these electrodes, indicating its largest specific capacitance (*C*_A_) due to the enlarged *S*_a_ under appropriate corrosion condition. For AAM1 and AAM2, pores grow unevenly and incompletely due to the insufficient corrosion time, resulting in the insufficient *S*_a_ and *C*_A_. For AAM4, AAM5, AAM6, prolonging corrosion time causes the pores to collapse while increasing the concentration of H_2_SO_4_ causes the corrosion to progress violently. Severe corrosion conditions would deteriorate the flexibility and supporting stress of Al foils, resulting in the decrease in *C*_A_. The CV curves demonstrate the largest *C*_A_ of AAM3 electrode due to the best etching modification of A3 foils, which are also consistent with the SEM images mentioned above. The resistivity of current collector is tested through a four-probe resistivity equipment (HPS2662, HELPASS). After coating the ultra-thin Au layer, the resistivity of the current collector drops from 0.41 Ω m^−1^ to 0.35 Ω m^−1^, indicating that the arming of Au layer improves the entire conductivity of current collector. The coating Au layer is formed of connected dots and dispersed uniformly to form a fast conductive path. Fig. S5† shows the physical and CV images of A3/MnO_2_ and AAM3 electrode. It is clearly that MnO_2_ can be hardly deposited on the A3 foil. Even the electrodeposition voltage is raised to 1.2 V, the MnO_2_ still cannot be deposited directly onto the Al foils. This may be due to the poor adhesion of MnO_2_ with aluminum oxide thin layer formed by oxidation on the surface of Al foil in the electrodeposition system. The greatly increased capacitance of AAM3 compared with A3/MnO_2_ electrode further confirms the necessity of the Au layer as a transition layer, which greatly enhances the adhesion between current collector and MnO_2_.

As the AAM3 electrode presents the largest CV loop and *C*_A_ among the obtained electrodes, the properties of AAM3 are further investigated in detail. Fig. 4b shows the CV curves of AAM3 measured from 5 mV s^−1^ to 100 mV s^−1^. The current density increases with scan rate. CV curves under low scan rates (5, 10, 25 mV s^−1^) present symmetric quasi-rectangular shapes without redox peaks, demonstrating the excellent storage characteristic and reversible pseudo-capacitance. The electrode tends to show a certain resistance behaviour and slightly deviates from the quasi-rectangular shapes at high scan rates (50, 100 mV s^−1^) due to the inefficient contact between MnO_2_ and electrolyte ions at high scan rates. In addition, the CV performance of electrodes obtained under different deposition voltages and times base on the A3/Au current collector are also investigate (Fig. S6†). It is found that the initial voltage that MnO_2_ can be successfully deposited onto the current collector is about 0.5 V, and deposition is hard to proceed for the voltage below this value. When the deposition voltage exceeds 0.8 V or the deposition time exceeds 400 s to further increase the mass loading of MnO_2_, the specific capacitance decreases instead. This phenomenon can be explained by the excessive MnO_2_ forming a thick layer that seals the nano-pores of the A3/Au current collector and prevents the internal MnO_2_ from effectively contacting the electrolyte, which retard the function of the porous structure.^15^

The rate capability of the AAM3 electrode is further investigated by GCD measurement with various current densities from 0.2 mA cm^−2^ to 6 mA cm^−2^ at a 0–0.8 V potential window (Fig. 4c). The GCD curves present nearly symmetric triangular shapes, showing the excellent electrochemical capacitance characteristics of AAM3 electrode. Besides, all GCD curves show well linearity in the relationship between potential and time, demonstrating the sufficient and reversible reaction between the MnO_2_ in electrode and the alkali ion (Na^+^) in electrolyte. The relationship between *C*_A_ and scan rate or current density is shown in Fig. 4d. The highest *C*_A_ calculated by CV curves achieves 219.3 mF cm^−2^ at 5 mV s^−1^, then gradually descends to 100.25 mF cm^−2^ at 100 mV s^−1^. The highest *C*_A_ evaluated by GCD curves achieves 222.13 mF cm^−2^ at 0.2 mA cm^−2^ or 302.9 F g^−1^ at 0.27 A g^−1^ (in order to compare with the Al/carbon electrode mentioned above). At low current density (0.2, 0.4 and 0.6 mA cm^−2^), the ions in the electrolyte can access and contact with almost all available active sites of MnO_2_, performing a complete insert-reaction and leading to high *C*_A_. However, this effective interaction between and MnO_2_ and electrolyte ions is hard to proceed thoroughly when increasing the current density, thus causing the reduction in *C*_A_. Nevertheless, even the current density is expanded by 30 times to 6 mA cm^−2^, the *C*_A_ can still maintain 139.5 mF cm^−2^, with a 63% retention, indicating excellent rate capability and rapid charge–discharge process. The excellent rate capability can be attributed to the nano-porous structure of metal/oxide electrode, in which nanoscale MnO_2_ grows along the internal pores and rough surface of highly conductive A3/Au current collector, allowing electrons to contact with electrolyte ions easily and efficiently to provide fast redox reaction even under a high current density. As shown in Table 1, the *C*_A_ of the AAM3 electrode is higher than comparable to the previous results of MnO_2_-based electrode. Unlike those MnO_2_-based electrodes mentioned in literatures, we start from the perspective of modification of Al/Au current collector, a relatively high specific capacitance can also be obtained without constructing complex multilayer structure or using expensive carbon-based materials or templates. It is believed that the AAM3 electrode stores charge by both the surface adsorption/desorption of Na^+^ cations and the reversible, and rapid MnO_2_ redox reaction (Mn^4+^/Mn^3+^) on the surface and interior of the electrode according to eqn (5) and (6).^4,43^5
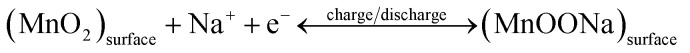
6
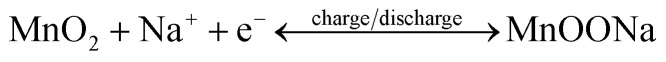


**Table 1:** Specific capacitance of some selected MnO_2_ based electrodes

Electrodes	Electrolyte	Specific capacitance (mF cm^−2^)	Operating voltage (V)	Ref.
MnO_2_/ZnO	1 M Na_2_SO_4_	230 at 10 mV s^−1^	0.8	11
MnO_2_@AuNF	1 M Na_2_SO_4_	8.26 at 5 mV s^−1^	0.8	34
Ni/rGO/MnO_2_	1 M Na_2_SO_4_	119.4 at 0.5 mA cm^−2^	0.8	35
α-Fe_2_O_3_/MnOx	1 M Na_2_SO_4_	227 at 0.5 mA cm^−2^	1.0	36
AgQDs/MnO_2_	1 M Na_2_SO_4_	97.4 at 0.02 mA cm^−2^	0.8	37
H-ZnO/MnO_2_	0.5 M N_a_2SO_4_	138.7 at 1 mA cm^−2^	0.8	38
FeOOH/MnO_2_	1 M LiClO_4_	252 at 1 mA cm^−2^	0.8	39
H-MnO_2_/CC	0.5 M Na_2_SO_4_	220 at 0.75 mA cm^−2^	0.8	40
MnO_2_ NWs	5 M LiCl	150 at 1 mA cm-^2^	0.8	41
CNPs/MnO_2_	0.1 M Na_2_SO_4_	109 at 5 mV s^−1^	0.8	42
AAM3	1 M Na_2_SO_4_	222.13 at 0.2 mA cm^−2^ or 219.3 at 5 mV s^−1^	0.8	This work

The frequency response and the conductivity of the AAM3 electrode are demonstrated by EIS measurement ranging from the frequency of 0.01 Hz to 100 kHz. Fig. 4e shows the Nyquist plots of the AAM3 electrode and the inset gives the equivalent circuit, in which *R*_s_, *C*_dl_, *R*_ct_, *Z*_w_ and *C*_L_ represents the intrinsic internal resistance,^44^ the double layer capacitance, the faradic charge-transfer resistance, the Warburg impedance, and the limit capacitance,^45^ respectively. The impedance spectrum exhibits a semicircle shape in the high frequency region and a straight line in the low frequency region. *R*_s_ and *R*_ct_ obtained from the high frequency region are about 3.26 Ω and 1.72 Ω, respectively, demonstrating the low internal and charge-transfer resistance due to the highly conductive A3/Au current collector. The slope of 45° portion of the curve reflects the *Z*_w_ of the electrode, representing fast ions diffusion from the electrolyte to the electrode.^37^ In addition, the straight line with a large slope in low frequency region indicates the ideal capacitance performance. The above results from Nyquist plots confirm the improved conductivity of coating Au layer upon A3 foil and the good adhesion between the current collector and MnO_2_, thus leading to the rapid ions diffusion/transportation ability at the interface of electrolyte/electrode and in the bulk electrode. Although MnO_2_ has inherently low conductivity which restricts its charge–discharge rate,^46^ the charge transfer reaction pseudocapacitance of the AAM3 electrode can be enhanced by rapid ions diffusion in the three-dimensional nano-porous current collector and through the highly conductive framework of the A3/Au foil as well as the metal/oxide interface.

The cyclic stability (Fig. 4f) of AAM3 is evaluated by GCD measurement for 2000 cycles under a current density of 3 mA cm^−2^. After cycling, the *C*_A_ of AAM3 decreases from 147 mF cm^−2^ to 125.25 mF cm^−2^, with 85.2% retention, showing prominent cyclic charge–discharge stability. It is observed that little MnO_2_ detach from the A3/Au current collector. The porous structure of A3/Au current collector can well withstand the volume expansion and contraction of MnO_2_ caused by cyclic charging and charging. The surface SEM images of the electrode after cycling is presented in Fig. S8.† It is found that the structure of MnO_2_ changes from nanowire to nanosheet after 2000 cycles (Fig. S8a and b†), leading to the decrease of specific capacitance. The AAM electrode prepared by the pristine Al/Au foil shows poor stability in the cyclic GCD test (Fig. S8c†), and *C*_A_ only remains 59.5% after 1000 cycles of test. After cyclic test, the MnO_2_ nanowires also convert to nanosheets, while some MnO_2_ film can be observed to delaminate from the Al/Au current collector (Fig. S8d†). This indicates that the MnO_2_ grows upon planar structure is easy to shed off when the volume changes, thereby leading to the degradation of *C*_A_ and poor stability.

### 3.3 Electrochemical properties and practicability of flexible symmetric solid-state supercapacitors

**Fig. 5:**
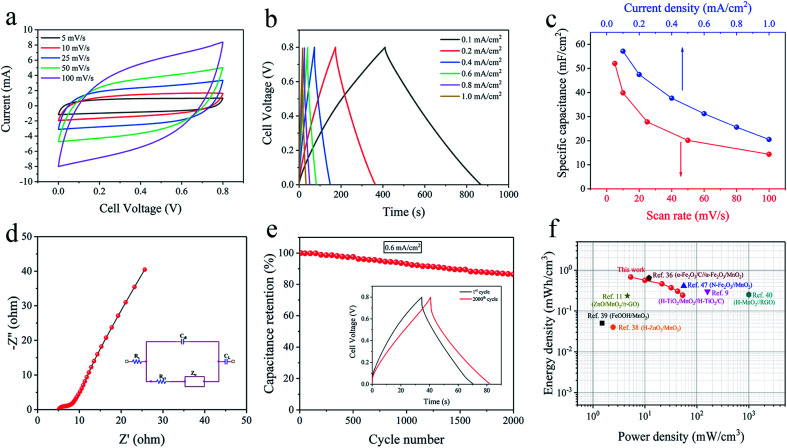
Electrochemical properties of the assembled solid-state supercapacitor under the voltage window of 0–0.8 V: (a) CV curves from 5 to 100 mV s^−1^, (b) GCD curves from 0.1 to 1 mA cm^−2^, (c) the relationship between the specific capacitance and the scan rate or current density, (d) Nyquist plots of the supercapacitor, (e) cyclic performance, (f) Ragone plots.

A symmetric solid-state supercapacitor was fabricated by two AAM3 electrodes face to face and Na_2_SO_4_/PVA gel as solid electrolyte. Further exploration of its electrochemical properties was performed under a two-electrode system. Fig. 5a displays the CV curves of the fabricated supercapacitor from 5 mV s^−1^ to 100 mV s^−1^ with a same voltage window (0–0.8 V). All CV curves present well symmetric and quasi-rectangular shape, indicating good capacitive behaviour of the device. The GCD curves measured under various current densities exhibit typical symmetric triangular shapes with low voltage drop (Fig. 5b), demonstrating excellent electrochemical capacity and fast charge–discharge process of the supercapacitor. Fig. 5c also reveals the relationship between *C*_A_ of the supercapacitor and scan rate or current density. The highest *C*_A_ achieves 52.07 mF cm^−2^ at 5 mV s^−1^ or 57.125 mF cm^−2^ at 0.1 mA cm^−2^, then descends to 14.36 mF cm^−2^ at 100 mV s^−1^ or 20.5 mF cm^−2^ at 1 mA cm^−2^, showing good rate capability. Under EIS measurement (Fig. 5d), this supercapacitor also presents a relatively low *R*_s_ of 5.12 Ω and *R*_ct_ of 3.24 Ω. After 2000 cycles GCD test under 0.6 mA cm^−2^ (Fig. 5e), the supercapacitor shows an 86.3% retention of *C*_A_, exhibiting impressive cyclic stability. The above superior properties of assembled supercapacitor can be ascribed to the combination of the nano-porous A3/Au skeleton and nano-MnO_2_, which allows more ions diffusion and faster electrons transportation at the interface of electrolyte/MnO_2_ and MnO_2_/current collector.

The Ragone plots (Fig. 5f) reveal the relationship between volumetric energy and power density of the assembled supercapacitor. The supercapacitor exhibits a maximum energy density of 0.68 mW h cm^−3^ at a power density of 5.33 mW cm^−3^, and a highest power density of 53.33 mW cm^−3^. These values exceed or are comparable to some previously reported symmetric systems based on MnO_2_, such as H-ZnO_2_/MnO_2_ (0.04 mW h cm^−3^),^38^ FeOOH/MnO_2_ (0.05 mW h cm^−3^),^39^ MnO_2_/CNPs (0.05 mW h cm^−3^)^42^ and some MnO_2_-based asymmetric supercapacitors, such as H-TiO_2_/MnO_2_//H-TiO_2_/C (0.30 mW h cm^−3^),^9^ ZnO/MnO_2_//r-GO (0.234 mW h cm^−3^),^11^ α-Fe_2_O_3_/C//α-Fe_2_O_3_/MnO_*x*_ (0.64 mW h cm^−3^),^36^ H-MnO_2_//RGO (0.25 mW h cm^−3^),^40^ α-Fe_2_O_3_ NTs//MnO_2_ NRs (0.55 mW h cm^−3^),^41^ N-Fe_2_O_3_//MnO_2_ (0.41 mW h cm^−3^)^47^ and MnO_2_/graphene//VOS@C (0.95 mW h cm^−3^).^48^ Moreover, based on the nano-porous current collector, different active materials can be grown to assemble into asymmetric supercapacitor, thus widening the charge–discharge voltage window and leading to significantly enhanced energy density. Further research is on the way.

**Fig. 6:**
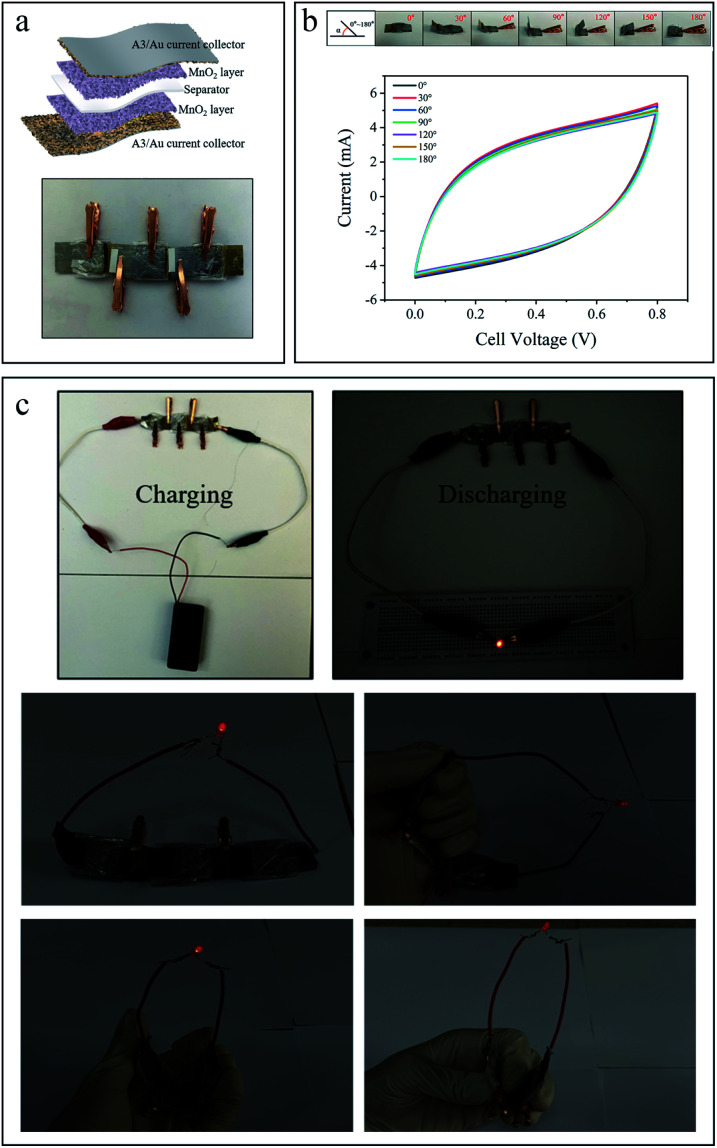
(a) Schematic drawing of the supercapacitor and physical drawing of three supercapacitors connected in series. (b) CV curves of the symmetric supercapacitor under different bending states at 50 mV s^−1^ within the voltage window of 0–0.8 V. (c) Images of three supercapacitors connected in series to charge and light up a red LED light under different bending states.

Fig. 6a shows the schematic drawing of the supercapacitor with a sandwich structure and the physical drawing of three supercapacitors connected in series. Furthermore, the flexibility of supercapacitor is demonstrated by CV measurement (Fig. 6b) under different bending states (from 0° to 180°) at the same scan rate of 50 mV s^−1^. Under different bending angles, the CV curves can well maintain the original quasi-rectangular shape with little change of capacity, indicating good mechanical stability of the device which can ascribe to the excellent flexibility of the Al current collector. The current collectors with qualified supportive capacity, flexibility and stability enable the supercapacitors have further possibilities in applications, such as micro and flexible electronic devices, which fully demonstrates the vital role of current collector in supercapacitors. In order to verify the practical value of the supercapacitor, three supercapacitors are connected in series firstly and then charged with two AA batteries for several seconds, then a commercial red LED light can be lit up under different bending states for a while (Fig. 6c). A switch is also added into the circuit, the supercapacitor after charging is used as the power source to control the LED on and off. The LED can response rapidly, which confirms the excellent stability and switching characteristic of the fabricated supercapacitors. The evidenced video is attached in the ESI.†

## 4. Conclusion

In summary, nano-porous Al/Au current collectors were developed by etching modification and ion-coating to synthesize Al/Au/MnO_2_ electrodes for high performance supercapacitors. This nano-porous skeleton with high specific surface area can support massive MnO_2_ to grow inside-out autonomously. Comparing with the electrode prepared by pristine Al foil, the modified Al/Au/MnO_2_ electrode exhibits greatly improved electrochemical performances, with a high areal specific capacitance of 222.13 mF cm^−2^ at 0.2 mA cm^−2^, superior rate capability and good cyclic stability. Besides, the assembled symmetric solid-state supercapacitor also presents a high specific capacitance of 57.13 mF cm^−2^, a high energy density of 0.68 mW h cm^−3^, and remarkable flexibility. The much-advanced properties can be attributed to the following reasons: (1) the highly conductive and nano-porous Al/Au current collector serves as an expressway for electrons transmission and easy access for electrolyte ions to the electrode; (2) the ultra-thin Au layer significantly improves the conductivity of the current collector and its adhesion with MnO_2_ in electrodeposition system; (3) nanoscale MnO_2_ prepared under appropriate electrodeposition condition can fully utilize the porous skeleton, therefore the MnO_2_ can contact fully with the electrolyte. Furthermore, the nano-porous Al/Au current collector is also able to support other active materials (NiO, PPy *etc.*) to prepare different electrodes and assemble into asymmetric supercapacitors. We hope that our study can provide an efficient method to prepare metal/oxide electrodes and attract more attention on the modification of current collector for high performance supercapacitor.

## Author contributions

Du Huang and Qian Xu: preparing the electrode and the supercapacitor, writing the first manuscript edition. Zhenya Lu: collecting the primary data and revising the manuscript. Xingyue Liu and Wenbin Yi: drawing figures and tables. Junning Gao, Zhiwu Chen, Xin Wang, Xiaoyi Fu: provide key suggestions for the experiment and the paper.

## Conflicts of interest

There are no conflicts to declare.

## Supplementary Material

RA-011-D1RA01923F-s001

RA-011-D1RA01923F-s002

RA-011-D1RA01923F-s003
