# Supplementary material for: Nano-porous Al/Au skeleton to support MnO2 with enhanced performance and electrodeposition adhesion for flexible supercapacitors
Source: RSC Adv. 2021 Jun 24;11(35):21405–13. doi: 10.1039/d1ra01923f (PMC9034163; doi:10.1039/d1ra01923f)
Supplement: RA-011-D1RA01923F-s003 [file RA-011-D1RA01923F-s003.pdf]

Electronic Supplementary Material (ESI) for RSC Advances.
This journal is © The Royal Society of Chemistry 2021

Nano-porous Al/Au skeleton to support MnO_2 with enhanced performance and electrodeposition adhesion for flexible supercapacitors

Qian Xu, Zhenya Lu*, Du Huang, Xingyue Liu, Wenbin Yi, Junning, Gao, Zhiwu Chen, Xin Wang, Xiaoyi Fu

School of Materials Science and Engineering, South China University of Technology, Guangzhou 510641, China.

* Corresponding Author E-mail Address: zhylu@scut.edu.cn

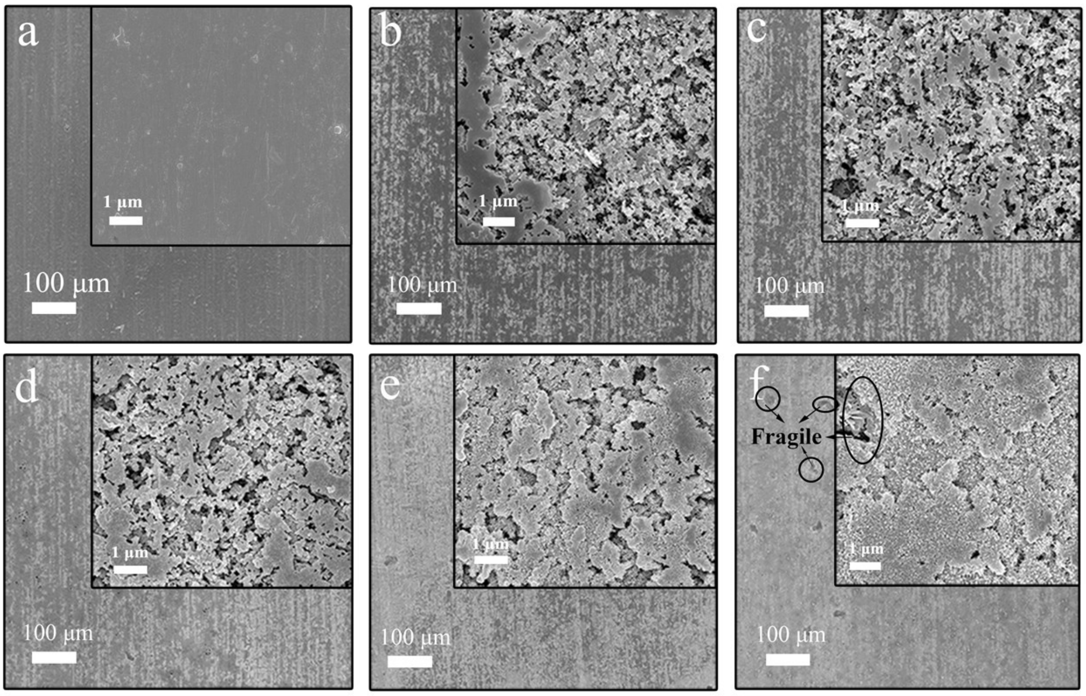

Figure S1. SEM images of pristine Al foil (a), A1(b), A2 (c), A4 (d), A5 (e), A6 (f).

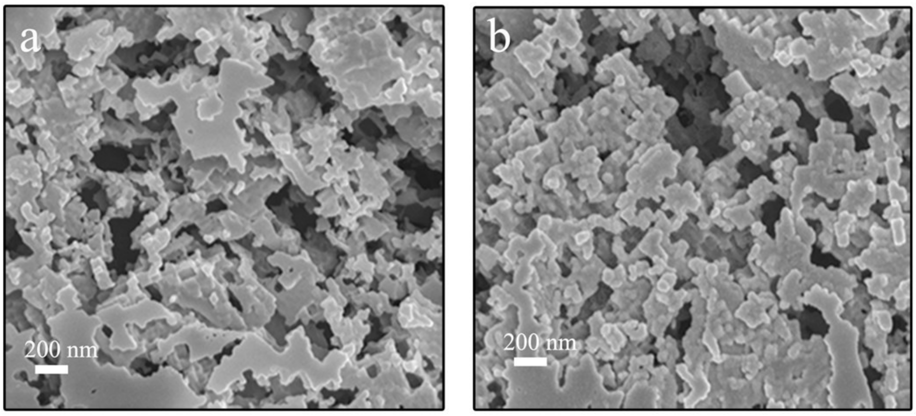

Figure S2. SEM images of (a) Al foils, (b) Al/Au foils.

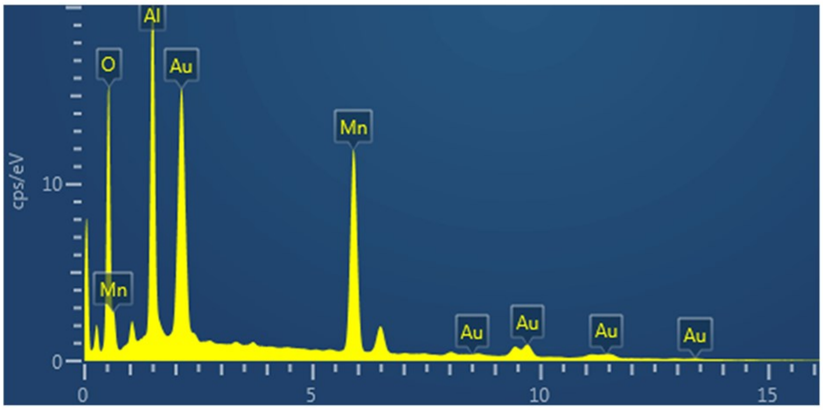

Figure S3. EDS spectrum of the AAM3 electrode.

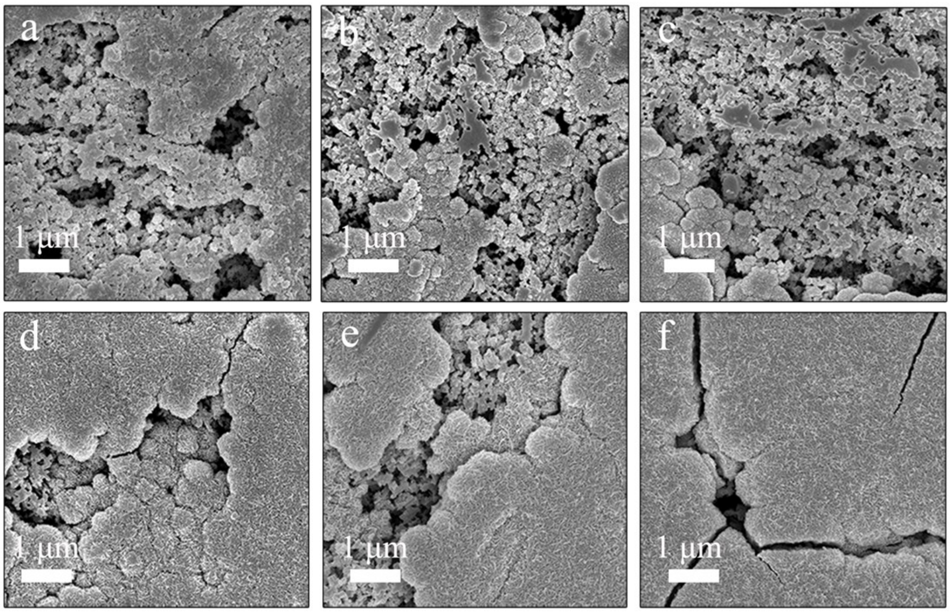

Figure S4. The SEM images of the electrodes obtained under the same electrodeposited voltage of + 0.8 V with different deposition time: (a) 10 s, (b) 20 s, (c) 50 s, (d) 100 s, (e) 200 s, (f) 300 s.

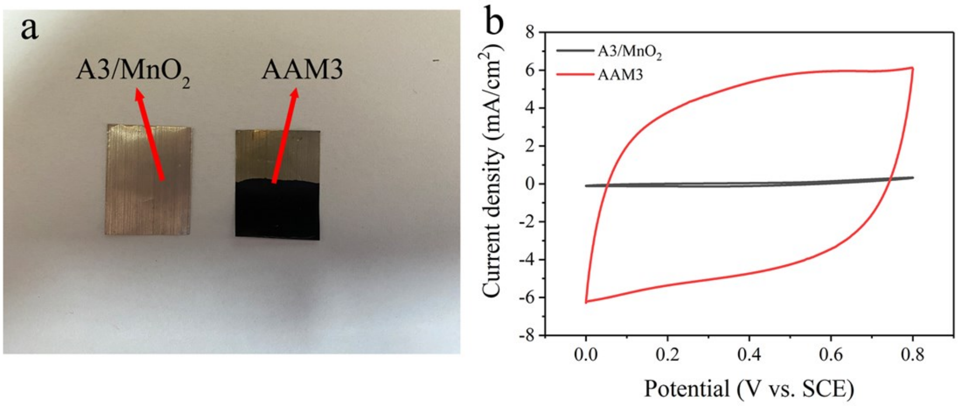

Figure S5. Physical and CV images of the A3/MnO_2 and the AAM3 electrode. Without Au layer, MnO_2 can hardly grow onto the Al current collector via electrodeposition.

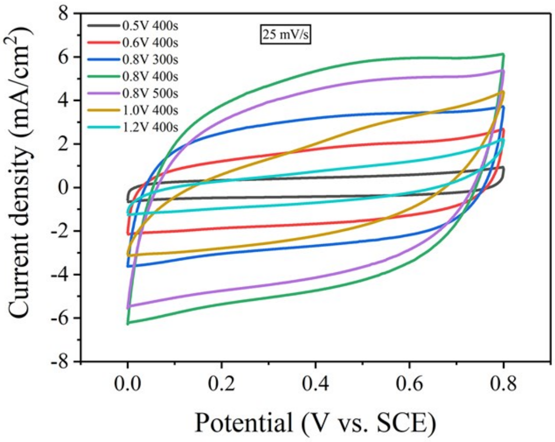

Figure S6. CV curves at 25 mV s^{-1} of the electrodes obtained under different deposition voltages and times based on A3/Au current collector.

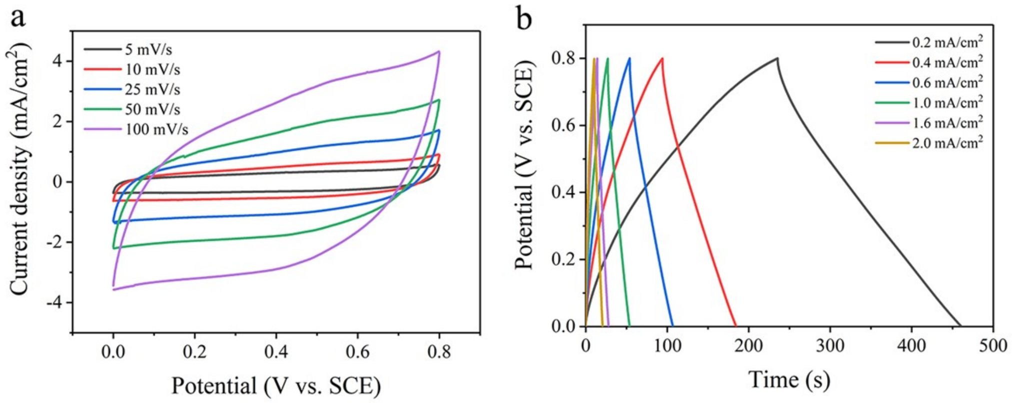

Figure S7. CV (a) and GCD (b) of the pristine AAM electrode.

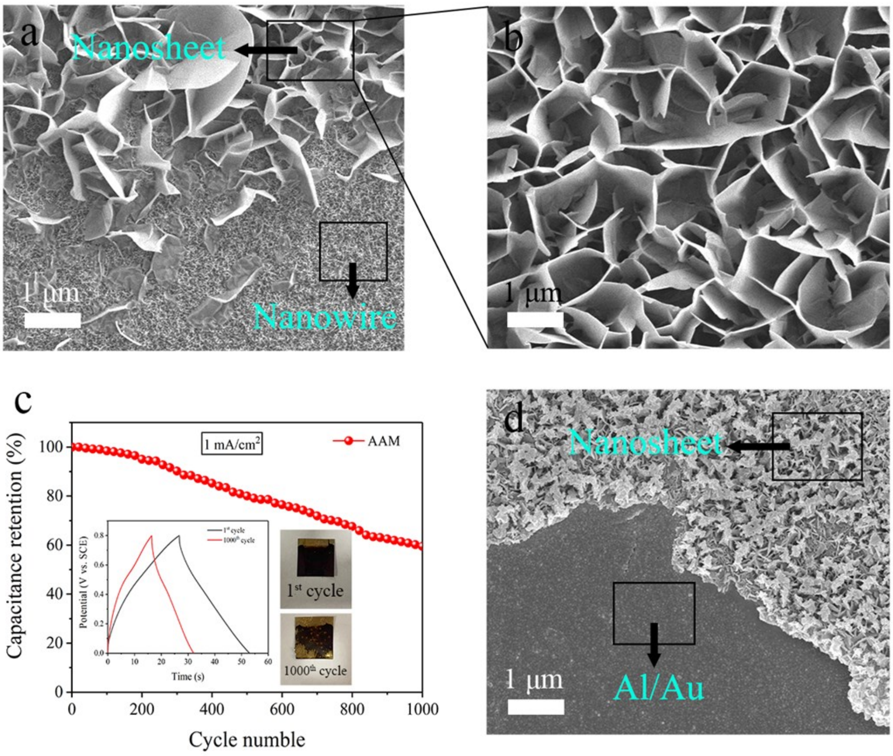

Figure S8. (a) The SEM image of the AAM3 electrode after cycling. (b) The enlarged SEM image of MnO_2 nanosheets. (c) The cyclic performance of the AAM electrode with the inset showing the GCD curves and the physical images of the first and the 1000th cycle at 1 mA cm^{-2}. (d) The SEM image of AAM electrode after cycling.
